# Improvement of Quality of Antenatal Care (ANC) Service Provision at the Public Health Facilities in Lao PDR: Perspective and Experiences of Supply and Demand Sides

**DOI:** 10.1186/s12884-019-2345-0

**Published:** 2019-07-22

**Authors:** Sysavanh Phommachanh, Dirk R. Essink, Maaike Jansen, Jacqueline E. W. Broerse, Pamela Wright, Mayfong Mayxay

**Affiliations:** 1 Institute of Research and Education Development, University of Health Sciences, Ministry of Health, Samsenthai Street, Pearvath Village, Sisathanark District, Vientiane Capital, Lao PDR P.O. Box: 7444,; 2 Vrije Universiteit Amsterdam, Athena Institute and Amsterdam Public Health Institute, Amsterdam, The Netherlands; 3 Guelph International Health Consulting, Amsterdam, The Netherlands; 4 Lao-Oxford-Mahosot Hospital-Wellcome Trust Research Unit (LOMWRU), Microbiology Laboratory, Mahosot Hospital, Vientiane, Lao PDR; 5 Centre for Tropical Medicine and Global Health, Nuffield Department of Clinical Medicine, University of Oxford, Oxford, UK

**Keywords:** Antenatal care (ANC), Quality improvement, Stakeholders’ perspectives, Supply and demand sides, Laos

## Abstract

**Background:**

The maternal mortality rate in Lao PDR (Laos) is still the highest in Southeast Asia, at 197 per 100,000 live births. Antenatal care (ANC) could contribute to maternal and child mortality reduction. The quality of ANC service remains inadequate and little information is available on the quality of health education and counseling services of health providers in Laos. This study aims to gain insight into the perceptions of stakeholders on both supply and demand sides of public ANC services in Laos and evidence for recommendations to improve the quality of ANC services.

**Methods:**

Semi-structured interviews were conducted with 50 participants from different stakeholder groups; on the demand side, couples with a currently pregnant woman and mothers with children under one year of age and a family member; and on the supply side, health providers, managers, policy makers of the Ministry of Health, and development partners. The interviews were voice recorded and transcribed verbatim for analysis by open and thematic coding, using the MAXQDA software program.

**Results:**

All respondents reported that the number of pregnant women who visit ANC services has increased. However, an analysis of the supply side identified issues related to the quality of ANC that need to be improved in the areas of facilities, human resources, privacy and confidentiality, providers’ behavior, attitudes, and ineffective communication skills when it comes to providing health education and counseling to pregnant women and their family members. The analysis of the demand side mainly emphasized the issues of providers’ behavior, attitude, communication and unequal treatment, and the lack of privacy. Both sides also suggested solutions to the problems, such as training, effective materials, rewarding good role models, and building a feedback system.

**Conclusion:**

The number of public ANC services has increased, but both supply and demand sides experienced challenges with the quality of ANC. All respondents proposed possible solutions to improve quality of ANC service in public health facilities in Laos.

## Background

Maternal and child mortality and morbidity are major health concerns worldwide [1]. Ninety-nine percent of deaths occur in low- and middle-income countries (LMICs), and almost one third in South Asia [1]. In the Lao People’s Democratic Republic (Lao PDR or Laos), the maternal mortality rate (MMR) is one of the highest in Asia at 197 deaths per 100,000 live births [2]. Like elsewhere in the world, the main causes of maternal death in Laos are post-partum bleeding, infections, pre-eclampsia and eclampsia, and other complications from delivery and from unsafe abortion [3]. The high MMR may be explained by the low coverage and inadequate quality of healthcare services.

The WHO prepared a framework for improvement of quality of care that requires effective provision of care and positive experience of care; it includes supply of competent, motivated staff and essential physical resources [4, 5]. A systematic review on effectiveness of strategies to improve health-care provider practices in LMICs suggested that multifaceted strategies can improve quality of health care service provision, such as health care provider-direct financial incentive, training, providing printed materials to be used during information and communication provision, monitoring, supervision, group solving problem in addition to strengthening infrastructure [6].

Effective pre-, intra-, and post-natal care interventions play an important role in reducing child mortality and morbidity [7–10]. Recent demographic household survey (DHS) data from 69 LMICs demonstrated that good ANC provision is directly associated with improved child health outcomes [11].

The quality of ANC services influences women’s healthcare seeking behavior [12, 13]. Effective communication skills would help to improve healthcare delivery [14]. Receiving good quality ANC is an important determinant of completing four or more ANC visits [15]. Poor quality of ANC created an obstacle for pregnant women to visit ANC [16] and also influenced good practice of mothers and family members [17].

In Laos, the quality of ANC provision in general remains inadequate. However, little is known about the health education and counseling given to clients during ANC consultation. Previous studies suggested that ANC quality was poor in rural areas due to the lack of equipment and materials and weak health care provider skills [18], and that deficiencies in qualified staff, basic supplies, budget and management were supply-side constraints, while demand-side constraints were related mainly to cost, limited access to transport, cultural practices and language [19]. These studies looked primarily into provider side perspectives and did not include ideas from pregnant women and mothers or other relevant stakeholders (such as family members, policy makers, and NGO staff). Including other types of respondents and broadening the geographic scope to include both rural and urban settings could provide a better understanding of where the weakest points lie in the quality of services.

This study aims to fill that gap, by looking into the quality of communication, specifically health education and counseling in the context of ANC in Laos. Stakeholders at all levels in the health system, from central to community, were invited to give their views. The specific objectives were 1) to explore the provision of care and experiences from supply and demand sides with reference to a quality of care framework that included routine care, providing information, effective communication, respect and dignity, emotional support, competence, motivation, and essential physical resources, and 2) to gain perspectives of different stakeholders on ways to improve the quality of ANC in public healthcare facilities in Laos. The results of this research could provide health policy makers and planners with insights from practice to improve the quality of ANC services particularly in the development and implementation of the new Lao ANC guidelines.

## Methods

### Study design and duration

This was a qualitative study with semi-structured interviews, conducted from April to July 2017 with key informants from different stakeholder groups using guidelines with open questions. At the same time as the interviews, we made on-the-spot observations of the healthcare facilities we were visiting.

### Key informants

We identified four stakeholder groups for this study. On the supply side: 1) directors and academic staff from each of the Department of Hygiene and Health Promotion, the Nutrition Centre, and the Information Education and Communication (IEC) Centre of the Ministry of Health (MoH) currently working in the area of nutrition, maternal and or child health; 2) health providers currently practicing at the ANC services, with at least one year’s experience in providing care to pregnant women, in the central, provincial, and district hospitals, as well as health centers; 3) representatives of development partners such Save the Children, WHO, UNICEF, UNFPA, Plan International, and World Food Program, staff currently working in the area of nutrition and/or maternal and child health; and on the demand side: 4) currently pregnant women, mothers with children under one year of age, and their family members (parent/husband/grandmother).

### Study site, sampling method and recruitment

**Fig. 1:**
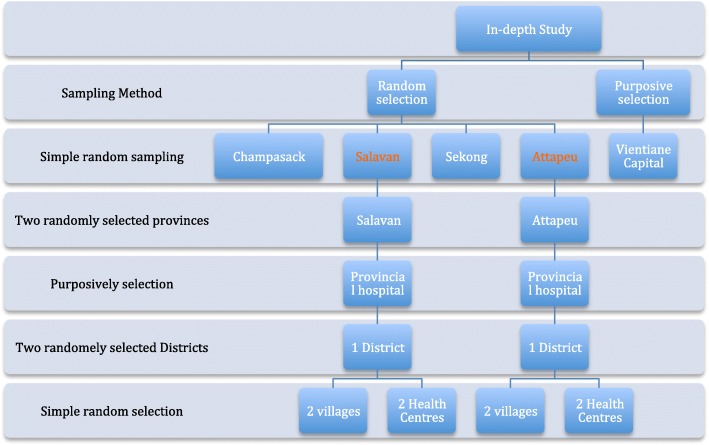
Study site and sampling methods. This figure shows study sites and sampling method. Vientiane Capital was purposively selected as the location of policy makers and development partners as well as the central level hospitals. Two of the four provinces (Champasack, Salavan, Sekong and Attapeu) in Southern Laos, were chosen by simple random selection to represent the area. Each province has a provincial hospital; several district hospitals and many health centers. Two villages and two health centers were randomly selected from each selected district

Study sites were selected using both purposive and random selection. Vientiane Capital was purposively selected as the location of policy makers and development partners as well as the central level hospitals. Of the four provinces (Champasack, Salavan, Sekong and Attapeu) in Southern Laos, where ANC services have been developed to a similar standard [20], two were chosen by simple random selection to represent the area, as a study site for this research and for a future intervention study (Fig. 1). Each province has a provincial hospital; several district hospitals and many health centers. In the provinces, data were collected from both health facilities and at community level in randomly selected villages.

Purposive sampling was applied to select key informants. We first made a list of desired key informants, sent them an official invitation letter and subsequently contacted them. In the provinces, the provincial health staff worked with local authorities to identify and invite key informants for the interview. Of the 56 key informants we planned to interview, 50 participated in the study. The supply side included five people working at the MoH, six from different development partners, six healthcare managers, and 17 healthcare providers. From the demand side, we interviewed eight couples (*n* = 16); four couples of Lao Loum group, which is the majority ethnic group in Laos (two couples with currently pregnant women and two couples with mothers having children under one year of age) and four couples of an ethnic minority group (two *Laven* and two *Brao (Lavae)* couples with currently pregnant women, and mothers with children under one year of age) (Table 1). The remaining six invited key informants did not participate because two of them no longer worked in ANC, while the other four were not available to be interviewed during the study period.

**Table 1:** Overview of key informants per level and province

Level	Stakeholder	Number
Subtotal of key informants in Vientiane Capital *N* = 22		
Subnational level	Policy maker and academic staff (Ministry of Health)	5
	Development partner	6
Central level	Health managers	2
	Health provider	9
Subtotal of key informants in Salavan Province *N* = 15		
Provincial level	Health manager	1
	Health provider	2
District level	Health manager	1
	Health provider	2
Health center	Health provider	1
Community level	Two Lao Loum couples (one with current pregnancy, one with mother with child under 1 year)	4
	Two couples of ethnic minority (one *Laven* with current pregnancy, one *Laven* of mother with child under 1 year)	4
Subtotal of key informants in Attapeu Province *N* = 13		
Provincial level	Health manager	1
	Health provider	1
District level	Health manager	1
	Health provider	1
Health center Level	Health provider	1
Community level	Two Lao Loum couples (one with current pregnancy, one of mother with child under 1 year)	4
	Two ethnic minority couples (one *Brao* with current pregnancy, one *Brao* of mother with child under 1 year)	4
Grand total interviews *N* = 50		
Subtotal interviews	Policy maker and academic staff of Ministry of Health	5
	Development partners	6
	Healthcare managers	6
	Health providers	17
	Service users or clients (demand side)	16

### Research tools

**Fig. 2:**
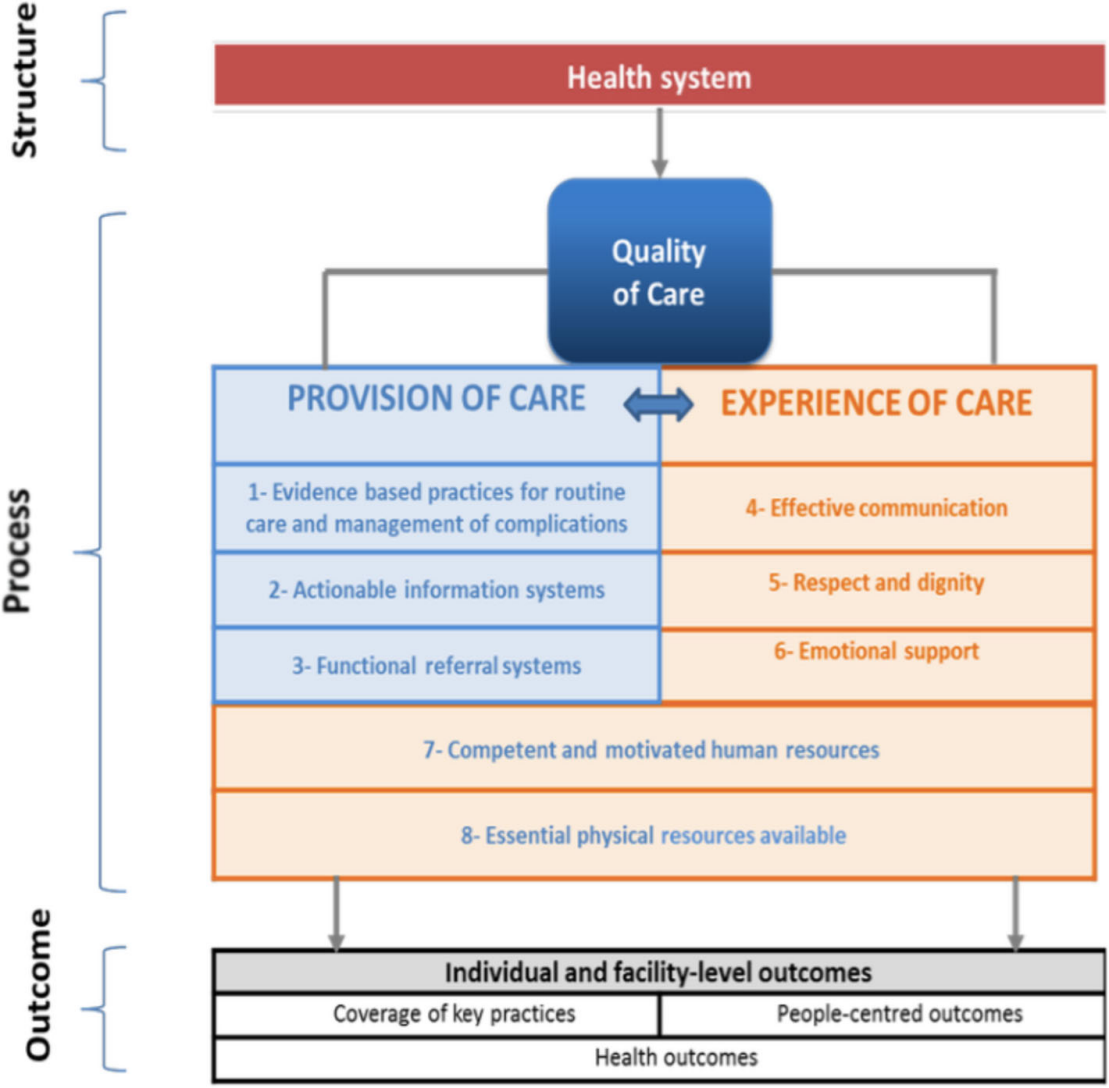
WHO Quality of Care Framework. This figure shows the concept of quality of care of WHO framework, and focusing on the provision of care and experience of care, such as, routine care, effective communication, respect-dignity, competent and motivation, and essential physical resources for this study. The data was analyzed using the concepts of the quality of care of WHO framework as mentioned above that all meaningful sentences were labeled based on the code tree using thematic coding and looking mainly at aspects of provision and experience of cares, staff competent, and materials

The interview guidelines were based on the concepts of the WHO Quality of Care Framework in 2015 [4], looking mainly at aspects of provision and experience of cares, staff competence, and materials, without looking into outcomes (see Fig. 2). The questions dealt mainly with the respondents’ experiences with the quality of ANC services, and what role they thought they could play to improve it. Different guides were used with different types of key informants.

### Semi-structure interviews (SSIs)

Data collection took place mostly at the participants’ office or in the homes of service users, where the participants would feel comfortable and privacy is ensured. The interviewers included one Lao academic researcher (SP), one expatriate Master student (MJ), and two field research assistants. The interviewers were provided with a three-day training course prior to data collection. The interview guides were piloted in the central and provincial levels with two MoH staff, four health providers and two mothers, which led to reformulation of several questions to finalize the guides.

Before the interview started, the interviewers explained the aim of the study and the general topics that would be discussed. They were told that the records would be anonymous and that they could withdraw at any moment without giving a reason. Written consent was obtained before each interview. Most of the providers were interviewed in English by MJ with the support of the local academic researcher (SP) who translated when needed. The service users were interviewed directly in the Lao language by SP. All interviews were recorded. The time of interviews ranged from 32 to 113 min (average 68). Notes were made while conducting the interviews, for the summary afterwards. During the interview, spot observational field notes were made which were included in the summary of the interview.

### Data analysis

The recordings and interview notes were used to generate verbatim written transcripts for the data analysis, which were analyzed using the software program MAXQDA. The research team read the transcripts a few times and discussed the open coding process together to reach consensus on the code tree. Then, using the concepts of the quality of care of WHO framework [4, 5] all meaningful sentences were labeled based on the code tree using thematic coding (see Fig. 2). Themes were considered significant where there was consistency across and within study participants and/or where they deepened understanding and captured something important in relation to the research question. The findings contain direct quotes from participants, which have been translated to English for clarity. After the data were analyzed, the findings were discussed with all researchers and supervisors to obtain consensus.

## Results

**Fig. 3:**
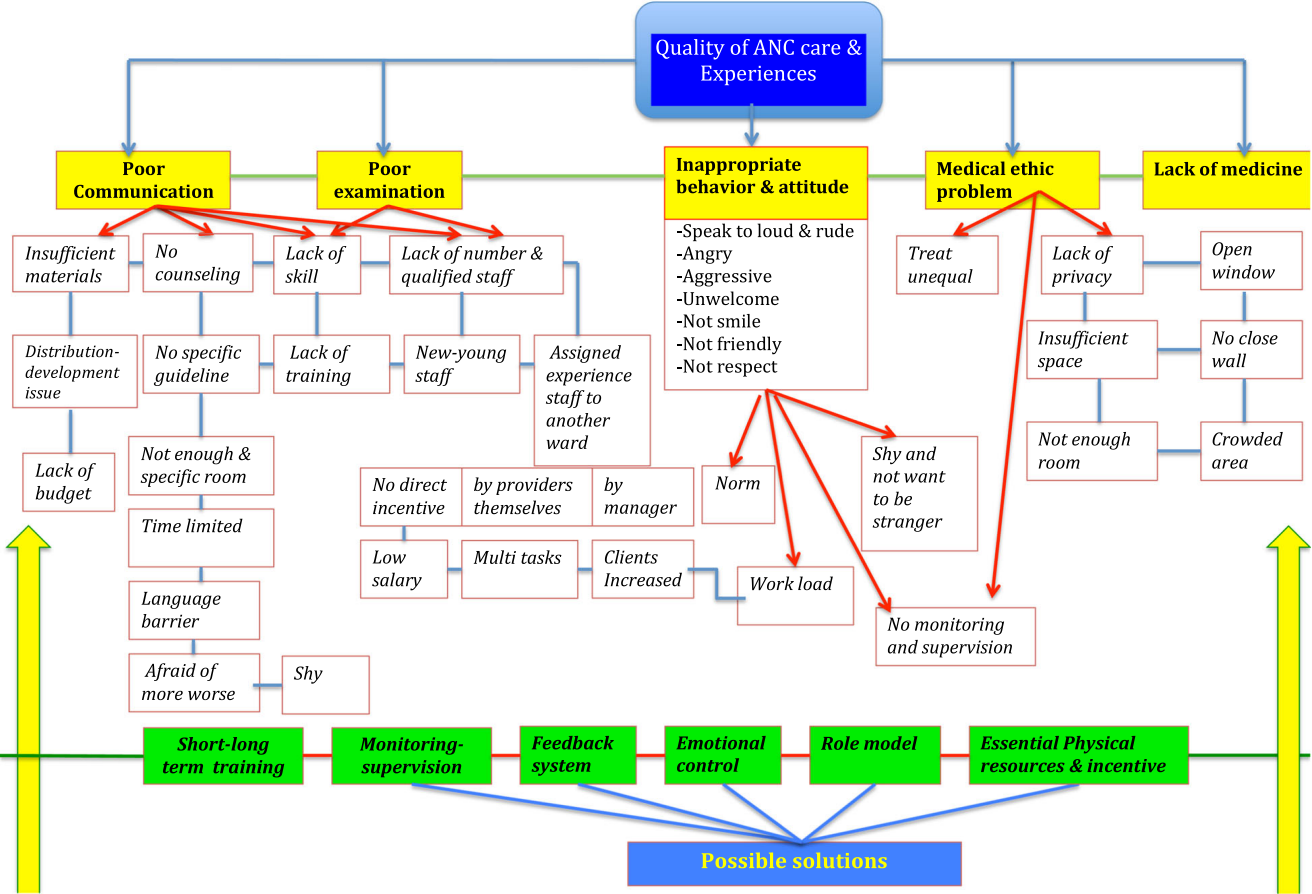
Problem tree of quality of ANC provision-experiences. This figure indicated the main problems of quality of ANC provision and experiences of care including possible solutions. The main problems included poor communication, poor examination, inappropriate behavior-attitude of health care providers, medical ethic issues, and lack of materials, lack of medicine and no specific room for counseling. However, the possible solutions for poor communication, poor examination, and medical ethic problem can be done by providing a short-term training for health care providers, and providing long-term training for students at medical school. In addition, providing monitoring-supervision, feedback system and role model would help to improve inappropriate behavior-attitude, and medical ethic problem. On the other hand, improving essential physical resources would help to solve the problems on lacking of essential medicines, effective material and specific room for counseling

The findings related to provision of care, experience of care, essential physical and human resources, staff competence, and outcomes, are described based on the concepts of quality of care as defined by WHO [4]. In Table 2 the first determinants in relation to the provision and experience of quality of ANC service are described, highlighting similarities and differences between supply and demand sides. Thereafter we discuss perspectives on possible solutions to improve the quality of ANC services (Fig. 3).

**Table 2:** Comparison of quality of ANC provision and experiences

Theme	Categories	Coding/concept/ theory	Description/definition/meaning	
			Supply side	Demand side
Challenges with quality of ANC service provision	Provision of care	Poor routine care	-Poor physical examination (they could not perform their task properly) -Did not provide medicine sometimes	▪ Not provide medicine ▪ Better not to come if do not get any medicine ▪ Asked to buy at drug store
	Experience of care	Poor information providing	Health providers could not provide sufficient information or provided very little information	▪ Providing too small information ▪ Never asked family member to listen
		Poor communication	-Only few minutes of time spending for individual health education and mostly during physical examination -Young staff were shy to talk during group health education -Language barrier for ethnic group -No counseling process -No training -No guideline/materials	▪ Providers talked very little without explanation ▪ They did not understand ▪ Family member know nothing ▪ Did not ask question
	Respect & dignity	No privacy and confidentiality	-Room needs to be shared with multiple women due to insufficient space -Overhear of conversation due to crowded area, open window, no closed wall -Providers did not ask permission before examination -Only few staff considered these issues as medical ethic problem).	▪ Often sharing the room ▪ Do not like other people to see their body and hear about their in formation ▪ Feel very ashamed ▪ Do not want to come if not necessary
		Treat unequally	Not mentioned/not perceived	▪ Providers paid more attention and treated better to the richer, relative, and or those who paid extra/additional money ▪ The poor hesitate to go to visit ANC afraid that no extra money to provide
		Inappropriate behaviour	Angry and aggressive	▪ Angry, aggressive, act as investigator, ordering advice, speak too load and very rude
		Negative attitude	-Bad mood due to work load, high pressure due to many clients with small staff, multiple duties, time limitation, very tiered, home stress -shy to talk very nice/soft voice, and do not want to be the only person who greeted to the women and family -Norm	▪ Unwelcome to patients ▪ Not friendly ▪ Not smile ▪ Bad mod sometimes ▪ Not polite
	Competent	Lack of qualified staff Lack of Skill	MCH trained staff were assigned to move to other wards -No training for new and young staff to provide care -Without training, some providers could not perform examination and providing information properly	▪ Not satisfy/happy with providers’ performance (eg. Performed very fast for physical examination, and did not tell anything
	Motivation	No intensive	-Low salary consideration -Designed by themselves to work at other wards where working based financing -No policy to support for motivation increment	▪ Not mention
	Essential physical resources	Insufficient space	-Not enough room, it needs to be shared during physical examination and providing information -Waiting area is very crowed	▪ Small room and not enough room, very crowded waiting areas
		Lack of staff	-Increasing number of visitors. -Not enough staff to provide care due to be assigned to move to work for other wards -Some decided by themselves to work for other wards where they can get incentive	▪ Sometimes could not see service providers at health facilities (in the community) ▪ Waiting very long time because of few staff perform their duties ▪
		Lack of material	-Insufficient health education materials -due to distribution problem and lack of budget at the lower level) -No specific IEC material to bring home	▪ Not use material for providing information ▪ No specific materials to bring home
		Lack of guideline	-No specific guideline to provide care at health facilities	▪ Not mentioned
		Lack of medicine	Not enough basic medicine in the routine care	▪ Not provide medicine, but also asked to buy at drug store

### Provision of care

The majority of participants mentioned that health providers could not always perform routine care correctly, which may be partly attributed to the lack of training, materials, and guidelines. For example, providers mentioned that (young) staff may have to perform their tasks without having had any specific training, so they lacked competence to measure the position and heartbeat of the fetus when performing obstetrical examination, or were not trained to provide health education on nutrition, physical activities, and danger signs for health care seeking during pregnancy and postpartum period, as illustrated by the quote below.

“*I think some young staff are not able to provide ANC service properly. They cannot identify the status of the fetus.”* (Participant No. 12, central level)

There were different understandings of how routine care should be provided, which can be seen from the diversity of guidelines observed as present in facilities. A few facilities developed their own clinical guidelines or SOPs, when a national standard guideline was not available. Another issue in routine care was a lack of essential medicines. For example, several respondents on the demand side mentioned that the health facilities had no medicine for them.

*“I was not very happy when I visited the health center. The doctor did not provide me any medicine, but told me to buy it outside at a drug store. So it is better to go to district hospital and provincial hospital.”* (Participant No. 1, community level)

### Experience of care

#### Effective health provider-service user communication

The respondents of both supply and demand sides alike mentioned that effective communication, especially in relation to counseling, was far from optimal. The main reasons given were similar as mentioned above: lack of time, guidelines, materials, and training. Counseling is not a stand-alone activity and is often given during physical examination. It was characterized by one-way provision of information rather than a mutual communication process. In some cases, health education was given to a group of several women and family members together at one time.

“*Some providers did not know how to explain properly to women and family members, due to lack of training and materials. I myself have never been trained for health education and counseling.”* (Participant No. 12, central level)

All health providers mentioned that time limitation did not allow them to provide sufficient information, because other clients were waiting outside. Some providers felt shy to speak with the women too much, because it is not their cultural habit to do so. Some providers did not ask the women about their needs, because they were afraid of not being able to fulfill the needs, which would be unacceptable for them. Service users also reported that very limited information was provided, and only to women – not to their family members, as illustrated by the following quote:

“*I only accompanied my wife to the hospital and just waited outside the room. When I asked my wife about what the doctor told her, she said that the doctor talked very little and quickly during the physical exam, then asked her to go out, and she did not know and could not remember what the doctor talked about.”* (Participant No. 16, community level)

In some situations, providers explained that it is not their culture to have a discussion. Women see the doctor as an authority that should tell them what to do because of their knowledge. In addition, some healthcare providers noticed that even if women did have the opportunity to ask a question, they remained silent. Also, although it does not seem to be a hierarchical issue, the clients are sometimes reluctant to discuss their health problems with health providers since they may be afraid that the provider may become angry with them if they ask (too many) questions.

*“Some clients may want to talk more, but sometimes they are afraid that if they talk more to the provider, he/she may not provide very good care to them. So they only talk about small issues even if they have a problem.”* (Participant No. 14, central level)

According to development partners, participants at the MoH and some providers, when women do not ask questions or explain their personal issues to the healthcare providers, the provider cannot give appropriate counseling to the needs of the client. The development partners and the participants from the MoH all assumed that this way of providing information would result in better outcomes for both mother and newborn.

*“The issue is that they do not do counseling, while counseling is needed to bridge the gap between telling someone what to do or having a slogan or poster, and actually helping them [with tailor made advice] to achieve change.”* (Participant No. 1, central level)

### Respect, dignity and emotional support

*Inadequate behavior and attitude* of health providers was described as reflecting the problems of poor respect, lack of dignity and emotional support for users of ANC. Most participants mentioned that negative interpersonal interactions between health providers and service users occurred at all health facilities. For example, some providers were aggressive, unwelcoming, impolite, and unfriendly, spoke too loudly, used rude words, and did not smile to women and family members. Some service users mentioned that they experienced inappropriate behavior and negative attitude of health providers at least one time when they accessed ANC services in their community.

*“I used to go for ANC at the health center; the staff blamed me that my body was unclean, smelly [tomen bor aph num bor? = smelly body, didn’t you take a bath?], and shouted at me that next time I must take a bath before coming. The second time, when I went to district hospital for my child’s vaccination, a health provider scolded me, asking “where were you when our staff went to provide vaccination in the village?” I was scared and sad; I am a poor person but do not wish to meet this kind of person.”* (Participant No. 14, community level)

Some participants of the supply side tried to explain the reasons for such inappropriate behavior and negative attitude of health providers. Poor working environment resulting in stress, fatigue, frustration and poor job satisfaction were mentioned. These were caused by high workloads with long working hours, lack of supportive supervision, and insufficient salaries without additional incentives. Most providers indicated that they and their colleagues do not always comply with behavioral norms, such as speaking in a soft voice and properly greeting clients. Clients indeed indicated that some providers talked very loudly and this was seen as very inappropriate.

Another issue was the *lack of privacy.* It was observed, and confirmed in interviews, that it is common that several women were examined in the same room at the same time, at all levels of health facilities. Several service users also mentioned that sharing a room for physical examination was not appropriate, and they felt very ashamed if other people saw their bodies.

*“I felt very ashamed during physical examination, because other women were in the same room with me, and the window was also open, so people outside the room might see my body.”* (Participant No. 1, community level)

Furthermore, people in waiting areas could easily overhear conversations held in the examination room. For example, the windows towards the waiting area were open, there were no closed walls between the different rooms of the ANC services, and in some facilities, and these services were provided in the waiting room itself.

Moreover, most service user respondents reported unequal treatment, mainly at the lower level of health facilities, as a challenge. For example, providers paid more attention and were more polite to their own relatives, to more affluent users and to those who paid extra money to them.

*“I hesitated to visit ANC because I was afraid of paying more money to the provider. If I did not pay additional money or bring something to the health care worker, she may not treat me as well as she did others, especially her own family, those she knows well, and the richer people.”* (Participant No. 9, community level)

### Essential physical resources and competency of staff

The majority of the respondents clearly felt that poor provision and experience of care is caused by a number of factors concerning weak physical and human resources, including staff numbers, staff competence, materials, medicines, and physical space. Supply-side participants suggested four reasons for lack of (competent) staff: 1) experienced staff was assigned to work at another ward; 2) qualified staff volunteers to move where better incentives are available; 3) experienced staff was retired, and 4) new staff lacks experience and specific training.

Supply-side participants also noted the lack of specific health education materials and standard guidelines to be used at health facilities at all levels, possibly explained by problems with financial support, distribution mechanisms and development. For example, many participants mentioned that materials were sent from central to provincial level, at government expense, but then the provincial level has to send it on to district hospitals and health centers without any financial support. So materials that should have been available did not reach health providers at the lower levels. Several participants, particularly IEC academic staff, reported that most existing IEC materials were developed with support from development partners or government. IEC centers themselves have no specific budget to develop specific materials and were not involved in the development of clinical guidelines.

### Outcomes

Although we did not specifically ask about health outcomes, most participants referred to outcome indicators when discussing the quality of care. According to most participants from the supply side, utilization of ANC services has recently increased:

*“Our service is better than it has ever been; we have the rooms and place to provide the ANC service. Also the number of pregnant women who visit the ANC increased recently in our hospital.” (*Participant No. 20, central level hospital)

However, some respondents, on supply and demand sides alike, stated that if the overall quality is not adequate, this increased utilization is not going to be sustainable.

### Suggestion to improve the quality of ANC provision

Abundant suggestions were made about how to improve the quality of ANC service with respect to inadequate provision of care, poor experience of care, and inadequate human and physical resources. The most relevant proposals are discussed based on the responses of the study participants and how these are linked to the problems noted above.

#### Short and long term-training

All participants on the supply side suggested that training would be the first option to improve the health staff competency and increase awareness of medical ethics. For example, short course training, particularly for new staff, on routine care and effective communication skills would help to refresh health providers’ knowledge and skills. Also they strongly recommended that refresher training on medical ethics should be done at least once a year for health providers at all levels, which might help to reduce the problems of lack of privacy/confidentiality and unequal treatment in the public health facilities. In addition, they also suggested that there is a need for more substantial, long-term training. For example, effective communication skills, particularly counseling skills and a comprehensive medical ethics course, should be integrated into the curriculum of medical schools, and offered as extra-curricular courses to providers, as one health manager said.

*“Besides integrating communication skill into the curriculum in medical school, I think we must continue to provide medical ethics training to health care providers, to repeat and remind them, because the existing curriculum of the university may not be complete.”* (Participant No. 19, central level).

#### Specific materials and standard guideline

Besides the training, nearly all participants on both supply and demand sides suggested that national standard guidelines and effective materials should be available to health providers at all levels, to help provide more effective ANC services with a constant standard. Participants of both sides specified that effective materials must be clear, with attractive pictures, for both health providers and service users. Many participants, the IEC staff in particular, proposed that the design of materials should involve the clients or health care users as well as providers, from community level up. Most participants also suggested providing specific material for clients to bring home to increase the knowledge of service users, and possibly to influence other people living in the same family and community. For example, many demand side participants mentioned that it would be good if women could be given effective materials that they could share with others, particularly husbands and elderly people who have more authority in their communities.

*“I think other pregnant women would also like to get very good material with clear and very nice pictures to bring home to tell family members; for myself, I would get more support from them about my health and about child care.”* (Participant No. 9, Community level)

#### Role model and peer-feedback system

All participants on the supply side mentioned that having good role models would be one of the most important ways to change health providers’ behavior and attitudes. For example, health providers could learn from and exchange experiences of best practices with other health providers, which might help them to gain more emotional control and increase confidence. Most participants on the supply side suggested that a good role model could be a person in their own health facilities, from other provinces, or even neighboring countries.

*“One way is to learn from colleagues abroad, specifically healthcare services in neighboring Thailand, which has similar culture and language, that would be the most effective way to increase health providers’ motivation to act differently.” (Participant No. 21, central level)*

Additionally, the majority of respondents strongly advised that one of the most important solutions, with low cost and simple implementation, would be a peer-feedback system, for example, a routine (weekly or monthly) feedback system among providers. There should also be individual feedback immediately by the manager. A feedback box should be more available and accessible for service users to give their feedback.

## Discussion

The study aimed to analyze the current quality of ANC provision within the public healthcare system in Laos and ways to improve it, with a focus on the need for good health education and counseling. This was done by exploring perspectives of a range of supply and demand side actors using the WHO’s Quality of Care Framework [4]. We found that both supply and demand sides experienced challenges with the inadequate quality of ANC provision, specifically poor routine care and ineffective communication, with inadequate behavior and attitude of healthcare providers. This was caused by insufficient quantity and quality of the supplies of essential physical resources, low staff competency, and low staff motivation.

Although ANC utilization has recently increased, there are serious issues with quality of care, which was, without hesitation, made clear by both health providers and policy makers and confirmed by reports of service users. The increase in use can likely be attributed to the recent policy implementation of *free delivery vouchers* for maternal and child health care [21, 22]. The increase in utilization may further compromise quality of care when the capacity of facilities is not increased. This in turn can lead to decreasing confidence in ANC services. The finding of this study also indicated that poor quality of ANC provision probably influenced clients’ ANC utilization, for example, clients did not wish to return to the ANC sites where they experienced unequal treatment and unwelcome behavior of health care providers, and did not receive medicines. It is essential to improve the quality of ANC service, because provision of good quality ANC services can have a great role in promoting utilization of health facilities. In Ethiopia, the provision of better quality of care increased the odds of giving birth at health facilities three times when compared to previously conventional practice [23]. In another study, regression analysis from a systematic review indicated that ANC with health facility delivery was positively correlated with safe delivery [24], however this systematic review suggested that multi-faceted strategies would be the best option to improve quality of health care services. For example, in addition to strengthening infrastructure, there should be sufficient support, especially providing incentives and training to health care providers, making printed materials for information and communication service available, performing regular monitoring and supervision. Based on the findings from our current study, we, therefore, strongly recommend that the policy makers/Lao government should invest more on those important items, to help improve the quality of ANC service at public health facilities in Laos.

The results indicated that essential physical and human resources remain poor in most health facilities at all levels. These findings are consistent with other research reports [18, 19, 25, 26] but as other studies focused mainly on issues of facilities and supplies, they did not report the key issues identified here, such as lack of privacy, respect and dignity underlying inadequate behavior and negative attitude of health providers. [18, 27]. Lack of essential physical and human resources, and lack of privacy while providing health care, are quite common in low and lower middle income countries [28, 29].

A lack of incentives for healthcare providers in their routine ANC service was identified as another major concern in most health facilities, which is found elsewhere, especially in China, where it was recently revealed that lack of incentives for health staff is a major concern, because they could not afford to increase payments for the staff [26]. A systematic review indicated that incentive provision has a positive effect on measures of quality of care [6]. Therefore the government may consider improving incentives (either in terms of money or other means such as certificate or medals acknowledging good performance or successful training) for health workers at public health facilities to increase motivation of health staff to perform their tasks more effectively.

In addition to inadequate job performance skills, the unavailability of materials and healthcare guidelines limited effective performance by health providers [30]. Having more effective communication skills would help to improve healthcare delivery [14]. Our findings suggest that communication skills of the providers remain poor due to the lack of training, IEC materials, and guidelines. There is a clear need to improve communication skills, including active listening, encouraging non-verbal communication, asking questions, giving clear explanations, clarifying and summarizing, being empathetic, providing feedback, as well as building trust and rapport [14].

Most respondents felt that poor staff competencies and the limited attention to medical ethical issues might be improved by training. The “Health Sector Reform Strategy and Framework” also stated that increasing staff competency and medical ethics through training helps to improve quality of care [21]. Taking a medical ethics training course could give health professionals the knowledge and skills to practice their mission in healthcare ethically [31]. In Laos, health providers are only trained on medical ethics at medical school as a requirement of the pre-medical course. The findings indicated that the medical ethics course at medical school may need to be revised or that refresher training would be needed for health providers at all levels.

Many study participants suggested that having role models of good practices is probably one good way to improve the interpersonal interactions between health providers and health users. Appropriate behavior and a positive attitude of health providers could become the normal practice at health facilities when health workers follow a good role model. Friendly, respectful and caring health providers can encourage health care utilization and client satisfaction [12]. It would be useful to identify good role models and to reward them in some way, to show others that their practice should be followed.

Although ANC service users mentioned that they experienced inappropriate behavior of health providers, both supply and demand sides might not be aware of the full potential of the negative effect of that problem. A study in Hong Kong demonstrated that emotional or psychological abuse during pregnancy resulted in a greater risk of postnatal depression, higher risk of thinking of harming themselves, and significantly poorer mental health-related quality of life [32]. Therefore, the feedback system in addition to the medical ethics course would probably also help to increase awareness of harmful practices and to improve inadequate behavior and negative attitudes of health providers. However, the feedback system should be available and accessible everywhere, as suggested by our respondents. Also, the skills of giving and receiving constructive feedback may not yet be common among the leaders and providers, so that would require training and advocacy as well.

It is well-known that a good quality of care plays an important role in reducing child mortality and morbidity [11]. Recent demographic household survey (DHS) data from 69 LMICs demonstrated that good ANC is directly associated with improved child health outcomes [11]; for example at least one visit to ANC by pregnant women was associated with reduced probability of both neonatal and infant mortality, while this probability was additionally reduced by having at least four ANC visits and having seen a skilled provider at least once [11]. The government should take action according to these findings, to improve the quality of ANC provision at public healthcare facilities in Laos aimed at reducing maternal and child mortality and morbidity.

Since this study was conducted within the framework of a research to policy program, the specific information needs for ANC guideline development were taken into account. Additionally, future medical ethics and communication trainings are planned to be developed and integrated into the curriculum of the University of Health Sciences, Lao PDR.

### Limitations

Since this qualitative study was only carried out in the two southern provinces of the country, we cannot generalize the findings to the whole country. However, according to the perceptions of the respondents from the central level, many issues arising in this study would be similar in other geographical areas of the country. Another issue is that some participants were interviewed in English with questions and answers translated back and forth between English and Lao, which could limit natural conversation between interviewers and interviewees. Respondents at community level were interviewed only in Lao, while some of them were from another ethnic group whose mother language was not Lao. However, dialect translation was done in some cases. All respondents appeared to express their opinions openly and recordings ensured that all information was collected for analysis.

## Conclusion

Although utilization of ANC services in Laos has recently increased, the quality of ANC service remains inadequate. The following actions should be taken by the Lao government: investing in the development of effective IEC materials for counseling and ANC guidelines; training on ANC routine care, counseling and medical ethics; increasing motivation for health care providers by providing direct incentives; and essential physical resources (medicine, equipment and better infrastructure). Additionally strengthening the feedback system and building exposure to good role models into both pre-service and in-service training are strongly recommended. Additional information to deepen understanding of the communication issues could be gained from a participant observation study and program implementation.

## Data Availability

The authors would like to confirm that the raw data of the manuscript can be provided with an appropriate request.

## Abbreviations

ANC: Antenatal Care
DHS: Demographic Household Survey
IEC: Information Education and Communication
Lao PDR: Lao People’s Democratic Republic
LMIC: Low- and Middle-Income Countries
MCNV: Medical Council Netherlands Vietnam
MMR: Maternal Mortality Rate
MOH: Ministry of Health
SOP: Standard Operation Procedure
SSI: Semi-structure Interview
UNFPA: United Nations Population Fund
UNICEF: United Nations International Children’s Emergency Fund
WHO: World Health Organization
